# Natural Language Processing for the Identification of Silent Brain Infarcts From Neuroimaging Reports

**DOI:** 10.2196/12109

**Published:** 2019-04-21

**Authors:** Sunyang Fu, Lester Y Leung, Yanshan Wang, Anne-Olivia Raulli, David F Kallmes, Kristin A Kinsman, Kristoff B Nelson, Michael S Clark, Patrick H Luetmer, Paul R Kingsbury, David M Kent, Hongfang Liu

**Affiliations:** 1 Department of Health Sciences Research Mayo Clinic Rochester, MN United States; 2 Department of Neurology Tufts Medical Center Boston, MA United States; 3 Department of Radiology Mayo Clinic Rochester, MN United States; 4 Institute for Clinical Research and Health Policy Studies Tufts Medical Center Boston, MA United States

**Keywords:** natural language processing, neuroimaging, electronic health records

## Abstract

**Background:**

Silent brain infarction (SBI) is defined as the presence of 1 or more brain lesions, presumed to be because of vascular occlusion, found by neuroimaging (magnetic resonance imaging or computed tomography) in patients without clinical manifestations of stroke. It is more common than stroke and can be detected in 20% of healthy elderly people. Early detection of SBI may mitigate the risk of stroke by offering preventative treatment plans. Natural language processing (NLP) techniques offer an opportunity to systematically identify SBI cases from electronic health records (EHRs) by extracting, normalizing, and classifying SBI-related incidental findings interpreted by radiologists from neuroimaging reports.

**Objective:**

This study aimed to develop NLP systems to determine individuals with incidentally discovered SBIs from neuroimaging reports at 2 sites: Mayo Clinic and Tufts Medical Center.

**Methods:**

Both rule-based and machine learning approaches were adopted in developing the NLP system. The rule-based system was implemented using the open source NLP pipeline MedTagger, developed by Mayo Clinic. Features for rule-based systems, including significant words and patterns related to SBI, were generated using pointwise mutual information. The machine learning models adopted convolutional neural network (CNN), random forest, support vector machine, and logistic regression. The performance of the NLP algorithm was compared with a manually created gold standard. The gold standard dataset includes 1000 radiology reports randomly retrieved from the 2 study sites (Mayo and Tufts) corresponding to patients with no prior or current diagnosis of stroke or dementia. 400 out of the 1000 reports were randomly sampled and double read to determine interannotator agreements. The gold standard dataset was equally split to 3 subsets for training, developing, and testing.

**Results:**

Among the 400 reports selected to determine interannotator agreement, 5 reports were removed due to invalid scan types. The interannotator agreements across Mayo and Tufts neuroimaging reports were 0.87 and 0.91, respectively. The rule-based system yielded the best performance of predicting SBI with an accuracy, sensitivity, specificity, positive predictive value (PPV), and negative predictive value (NPV) of 0.991, 0.925, 1.000, 1.000, and 0.990, respectively. The CNN achieved the best score on predicting white matter disease (WMD) with an accuracy, sensitivity, specificity, PPV, and NPV of 0.994, 0.994, 0.994, 0.994, and 0.994, respectively.

**Conclusions:**

We adopted a standardized data abstraction and modeling process to developed NLP techniques (rule-based and machine learning) to detect incidental SBIs and WMDs from annotated neuroimaging reports. Validation statistics suggested a high feasibility of detecting SBIs and WMDs from EHRs using NLP.

## Introduction

### Background

Silent brain infarction (SBI) is defined as the presence of 1 or more brain lesions, presumed to be because of vascular occlusion, found by neuroimaging (magnetic resonance imaging, MRI or computed tomography, CT) in patients without clinical manifestations of stroke. SBIs are more common than stroke and can be detected on MRI in 20% of healthy elderly [1-3]. Studies have shown that SBIs are associated with increased risk of subsequent stroke, cognitive decline, and deficiency in physical function [1,2]. Despite the high prevalence and serious consequences, there is no consensus on the management of SBI as routinely discovering SBIs is challenged by the absence of corresponding diagnosis codes and the lack of the knowledge about the characteristics of the affected population, treatment patterns, or the effectiveness of therapy [1]. Even though there is strong evidence shows that antiplatelet and statin therapies are effective in preventing recurrent stroke in patients with prior stroke, the degree to which these results might apply to patients with SBI is unclear. Although SBI is understood by some clinicians to be pathophysiologically identical to stroke (and thus similarly treated), others view SBI as an incidental neuroimaging finding of unclear significance. The American Heart Association/American Stroke Association has identified SBI as a major priority for new studies on stroke prevention because the population affected by SBI falls between primary and secondary stroke prevention [4].

In addition to SBI, white matter disease (WMD) or leukoaraiosis is another common finding in neuroimaging of elderly. Similar to SBI, WMD is usually detected incidentally on brain scans and is commonly believed to be a form of microvascular ischemic brain damage resulting from typical cardiovascular risk factors [5]. WMD is associated with subcortical infarcts due to small vessel disease and is predictive of functional disability, recurrent stroke, and dementia [6-8]. SBI and WMD are related, but it is unclear whether they result from the same, independent, or synergistic processes [9,10]. As with SBI, there are no proven preventive treatments or guidelines regarding the initiation of risk factor–modifying therapies when WMD is discovered.

### Objectives

Identifying patients with SBI is challenged by the absence of corresponding diagnosis codes. One reason is that SBI-related incidental findings are not included in a patient’s problem list or other structured fields of electronic health records (EHRs); instead, the findings are captured in neuroimaging reports. A neuroimaging report is a type of EHR data that contains the interpretation and finding from neuroimage such as CT and MRI in unstructured text. Incidental SBIs can be detected by the review of neuroradiology reports obtained in clinical practice, typically performed manually by radiologists or neurologists. However, manually extracting information from patient narratives is time-consuming, costly, and lacks robustness and standardization [11-14]. Natural language processing (NLP) has been leveraged to perform chart review for other medical conditions by automatically extracting important clinical concepts from unstructured text. Researchers have used NLP systems to identify clinical syndromes and biomedical concepts from clinical notes, radiology reports, and surgery operative notes [15]. An increasing amount of NLP-enabled clinical research has been reported, ranging from identifying patient safety occurrences [16] to facilitating pharmacogenomic studies [17]. Our study focuses on developing NLP algorithms to routinely detect incidental SBIs and WMDs.

## Methods

### Study Setting

This study was approved by the Mayo Clinic and Tufts Medical Center (TMC) institutional review boards. This work is part of the Effectiveness of Stroke PREvention in Silent StrOke project, which is to use NLP techniques to identify individuals with incidentally discovered SBIs from radiology reports, at 2 sites: Mayo Clinic and TMC.

### Gold Standard

The detailed process of generating the gold standard is described in Multimedia Appendix 1. The gold standard annotation guideline was developed by 2 subject matter experts: a vascular neurologist (LYL) and a neuroradiologist (PHL), and the annotation task was performed by 2 third-year residents (KAK, MSC) from Mayo and 2 first-year residents (AOR, KN) from TMC. Each report was annotated with 1 of the 3 labels for SBI (positive SBI, indeterminate SBI, or negative SBI) and one of the 3 labels for WMD (positive WMD, indeterminate WMD, or negative WMD).

The gold standard dataset includes 1000 radiology reports randomly retrieved from the 2 study sites (500 from Mayo Clinic and 500 from TMC) corresponding to patients with no prior or current diagnosis of stroke or dementia. To calculate interannotator agreement (IAA), 400 out of the 1000 reports were randomly sampled and double read. The gold standard dataset was equally split to 3 subsets for training (334), developing (333), and testing (333).

### Experimental Methods

We compared 2 NLP approaches. One was to define the task an information extraction (IE) task, where a rule-based IE system can be developed to extract SBI or WMD findings. The other was to define the task as a sentence classification task, where sentences can be classified to contain SBI or WMD findings.

#### Rule-Based Information Extraction

**Figure 1:**
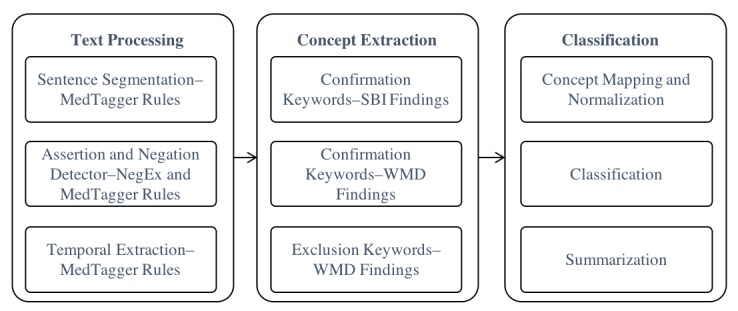
Rule system process flow. SBI: silent brain infarction; WMD: white matter disease.

We adopted the open source NLP pipeline, MedTagger, as the infrastructure for the rule-based system implementation. MedTagger is a resource-driven, open source unstructured information management architecture–based IE framework [18]. The system separates task-specific NLP knowledge engineering from the generic NLP process, which enables words and phrases containing clinical information to be directly coded by subject matter experts. The tool has been utilized in the eMERGE consortium to develop NLP-based phenotyping algorithms [19]. Figure 1 shows the process workflow. The generic NLP process includes sentence tokenization, text segmentation, and context detection. The task-specific NLP process includes the detection of concept mentions in the text using regular expressions and normalized to specific concepts. The summarization component applies heuristic rules for assigning the labels to the document.

For example, the sentence “probable right old frontal lobe subcortical infarct as described above,” is processed as an SBI concept with the corresponding contextual information with status as “probable,” temporality as “present,” and experiencer as “patient.”

The domain-specific NLP knowledge engineering was developed following 3 steps: (1) Prototype algorithm development, (2) Formative algorithm development using the training data, and (3) Final algorithm evaluation. We leveraged pointwise mutual information [20] to identify significant words and patterns associated with each condition for prototyping the algorithm (Multimedia Appendix 2). The algorithm was applied to the training data. False classified reports were manually reviewed by 2 domain experts (LYL, PHL). Keywords were manually curated through an iteratively refining process until all issues were resolved. The full list of concepts, keywords, modifiers, and diseases categories are listed in Textbox 1.

**Silent brain infarction (SBI) and white matter disease (WMD) risk factor and indication keywords.**

- Confirmation keywords—disease-finding SBI: infarct, infarcts, infarctions, infarction, lacune, lacunes
- Confirmation keywords—disease modifier SBI: acute, acute or subacute, recent, new, remote, old, chronic, prior, chronic foci of, benign, stable small, stable
- Confirmation keywords—disease location SBI: territorial, lacunar, cerebellar, cortical, frontal, caudate, right frontoparietal lobe, right frontal cortical, right frontal lobe, embolic, left basal ganglia lacunar, basal ganglia lacunar, left caudate and left putamen lacunar
- Confirmation keywords—disease-finding WMD: leukoaraiosis, white matter, microvascular ischemic, microvascular leukemic, microvascular degenerative
- Exclusion WMD: degenerative changes

#### Machine Learning

**Figure 2:**
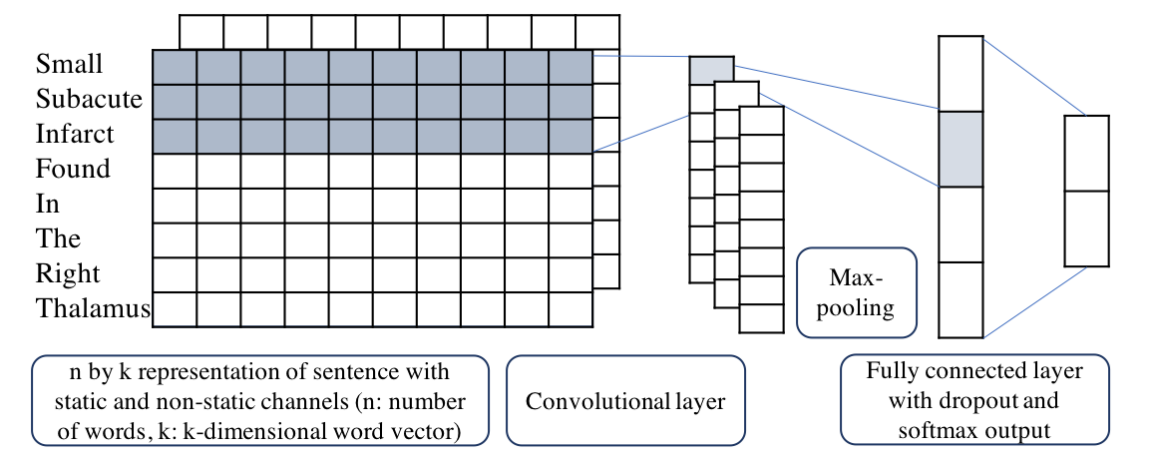
Convolutional neural network architecture with 2 channels for an example sentence.

The machine learning (ML) approach allows the system to automatically learn robust decision rules from labeled training data. The task was defined as a sequential sentence classification task. We adopted Kim’s convolutional neural network (CNN) [21] and implemented using TensorFlow 1.1.02 [22]. The model architecture, shown in Figure 2, is a variation of the CNN architecture of Collobert R [23].

We also adopted 3 traditional ML models—random forest [24], support vector machine [25] and logistic regression [26]—for baseline comparison. All models used word vector as input representation, where each word from the input sentence is represented as the k-dimensional word vector. The word vector is generated from word embedding, a learned representation for text where words that have the same meaning have a similar representation. Suppose x_1_, x_2_, … , x_n_ is the sequence of word representations in a sentence where

x_i_ = E_xi_, I = 1,2, …, n.

Here, E_xi_ is the word embedding representation for word x_i_ with the dimensionality d. In our ML experiment, we used Wang’s word embedding trained from Mayo Clinic clinical notes where d=100 [27]. The embedding model is the skip-gram of word2vec, an architecture proposed by Mikolov T [28]. Let x_i:i+k-1_ represent a window of size k in the sentence. Then the output sequence of the convolutional layer is

con_i_ = f(w_k_ x_i:i+k-1_ + b_k_),

where f is a rectify linear unit function, w_k_ and b_k_ are the learning parameters. Max pooling was then performed to record the largest number from each feature map. By doing so, we obtained fixed length global features for the whole sentence, that is,

m_k_ = max_1≤i≤n-k+1_(con_i_).

Then the features are fit into a fully connected layer with the output being the final feature vector O=wm_k_ + b. Finally, a softmax function is utilized to make final classification decision, that is,

p(sbi│x,θ) = e^(O_sbi_)/(e^(O_sbi_)+e^(O_other_)),

where θ is a vector of the hyper parameters of the model, such as w_k_, b_k_, w and b.

### Evaluation Metric

For evaluation of the quality of the annotated corpus, Cohen kappa was calculated to measure the IAA during all phases [29]. As the primary objective of the study is case ascertainment, we calculated the IAA at the report level.

A 2 x 2 confusion matrix was used to calculate performance score for model evaluation: positive predictive value (PPV), sensitivity, negative predictive value (NPV), specificity, and accuracy using manual annotation as the gold standard. The McNemar test was adopted to evaluate the performance difference between the rule-based and ML models [30,31]. To have a better understanding of the potential variation between neuroimaging reports and neuroimages, we compared the model with the best performance (rule-based) with neuroimaging interpretation. A total of 12 CT images and 12 MRI images were stratified—randomly sampled from the test set. A total of 2 attending neurologists read all 24 images and assigned the SBI and WMD status. The cases with discrepancies were adjudicated by the neuroradiologist (PHL) The agreement was assessed using kappa and F-measure [32].

## Results

### Interannotator Agreements Across Neuroimaging Reports

Among the total 400 double-read reports, 5 reports were removed because of invalid scan types. The IAAs across Mayo and Tufts neuroimaging reports were 0.87 and 0.91. Overall, there is a high agreement between readers on both reports (Tables 1 and 2). Age-specific prevalence of SBI and WMD is provided in Multimedia Appendix 2.

**Table 1:** Interreader agreement across 207 Mayo neuroimaging reports.

Interannotator agreement	Computed tomography (n=63)		Magnetic resonance imaging (n=144)		Total (n=207)	
	% agree	kappa	% agree	kappa	% agree	kappa
Silent brain infarction	98.4	0.92	97.2	0.83	97.6	0.87
White matter disease	100.0	1.00	98.6	0.97	99.0	0.98

**Table 2:** Interreader agreement across 188 Tufts Medical Center neuroimaging reports.

Interannotator agreement	Computed tomography (n=80)		Magnetic resonance imaging (108)		Total (n=188)	
	% agree	kappa	% agree	kappa	% agree	kappa
Silent brain infarction	98.8	0.79	99.1	0.94	99.5	0.91
White matter disease	100.0	1.00	99.1	0.98	99.5	0.99

### Natural Language Processing System Performance

Overall, the rule-based system yielded the best performance of predicting SBI with an accuracy of 0.991. The CNN achieved the best score on predicting WMD (0.994). Full results are provided in Table 3.

According to the McNemar test, we found the difference between rule-based system and CNN on SBI is considered to be statistically significant (*P* value=.03). We found no statistically significant difference between the rest of the models.

Table 4 lists the evaluation results of NLP and gold standard derived from reports against the neuroimaging interpretation for SBI and WMD. Both NLP and gold standard had moderate-high agreements with the neuroimaging interpretation, with kappa scores around .5. Our further analysis showed the practice graded findings (gold standard and NLP) achieved high precision and moderate recall scores compared with the neuroimaging interpretation. Through the confirmation with Mayo and TMC radiologists, we believed such discrepancy was because of the inconsistency in documentation standards related to clinical incidental findings, causing SBIs and WMDs underreported.

**Table 3:** Performance on test dataset against human annotation as gold standard.

Evaluation of natural language processing, model name		Sensitivity	Specificity	Positive predictive value	Negative predictive value	Accuracy
**Silent brain infarction (n=333)**						
	Rule-based system	0.925	1.000	1.000	0.990	0.991
	CNN^a^	0.650	0.993	0.929	0.954	0.952
	Logistic regression	0.775	0.983	0.861	0.970	0.958
	SVM^b^	0.825	1.000	1.000	0.977	0.979
	Random forest	0.875	1.000	1.000	0.983	0.986
**White matter disease (n=333)**						
	Rule-based system	0.942	0.909	0.933	0.921	0.928
	CNN	0.994	0.994	0.994	0.994	0.994
	Logistic regression	0.906	0.865	0.896	0.877	0.888
	SVM	0.864	0.894	0.917	0.830	0.877
	Random forest	0.932	0.880	0.913	0.906	0.910

^a^CNN: convolutional neural network.

^b^SVM: support vector machine.

**Table 4:** Comparison of the neuroimaging interpretation with gold standard and natural language processing.

Evaluation of natural language processing against the neuroimaging interpretation		F-measure	kappa	Precision	Recall
**Silent brain infarction (n=24)**					
	Gold standard	0.74	0.50	0.92	0.69
	NLP^a^	0.74	0.50	0.92	0.69
**White matter disease (n=24)**					
	Gold standard	0.78	0.56	0.86	0.80
	NLP	0.74	0.49	0.85	0.73

^a^NLP: natural language processing.

## Discussion

### Machine Learning Versus Rule

In summary, the rule-based system achieved the best performance of predicting SBI, and the CNN model yielded the highest score of predicting WMD. When detecting SBI, the ML models were able to achieve high specificity, NPV, and PPV but moderate sensitivity because of the small number of positive cases. Oversampling is a technique to adjust the class distribution of training data to balance the ratio between positive and negative cases [33]. This technique was applied to the training data to help boost the signals of positive SBIs. The performance was slightly improved but was limited by the issue of overfitting, a situation when a model learns the training data too well. Due to that, unnecessary details and noises in the training data can create negative impact to the generalizability of the model. In our case, the Mayo reports have larger language variation (noise) because of a free style of documentation method, whereas TMC uses a template-based documentation method. According to the sublanguage analysis, Mayo had 212 unique expressions for describing no acute infarction, whereas TMC had only 12. Therefore, the model trained on oversampled data had a bias toward the expressions that only appeared in the training set. When predicting WMD, the ML model outperformed the rule-based model. The reason is because the dataset for WMD is more balanced than SBI (60% positive cases), which allows the system to equally learn from both classes (positive and negative). The overall performance on WMD is better than SBI because WMDs are often explicitly documented as important findings in the neuroimaging report.

### False Prediction Analysis

Coreference resolution was the major challenge to the rule-based model for identifying SBIs. Coreference resolution is an NLP task to determine whether 2 mentioned concepts refer to the same real-world entity. For example, in Textbox 2, “The above findings” refers to “where there is an associated region of nonenhancing encephalomalacia and linear hemosiderin disposition.” To determine if a finding is SBI positive, the system needs to extract both concepts and detect their coreference relationship.

**Example of coreference resolution.**

“Scattered, nonspecific T2 foci, most prominently in the left parietal white matter *<Concept 1>where there is an associated region of nonenhancing encephalomalacia and linear hemosiderin disposition. <Concept 1/>* Linear hemosiderin deposition overlying the right temporal lobe (series 9, image 16) as well. No abnormal enhancement today. *<Concept 2>The above findings are nonspecific but the evolution, hemosiderin deposition, and gliosis suggest post ischemic change. <Concept 2>*”

For the ML system, the false positives from the identification of SBIs were commonly contributed by disease locations. As the keywords *foci, right occipital lobe, right parietal lobe, right subinsular region*, and *left frontal region* often coexisted with SBI expressions, the model assigned higher weights to these concepts when the model was trained. For example, the expression: “there are a bilateral intraparenchymal foci of susceptibility artifact in the right occipital lobe, right parietal lobe, right subinsular region and left frontal region” has 4 locations with no mention of “infarction” appearing in the sentence. The ML system still predicted it as SBI positive. Among all ML models, the CNN yielded the worse NPV, which suggested the CNN was more likely to receive false signals from disease locations. Our next step is to further refine the system by increasing the volume of training size through leveraging distant supervision to obtain additional SBI positive cases.

### Limitations

Our study has several limitations. First, despite the high feasibility of detecting SBIs from neuroimaging reports, there is a variation between NLP-labeled neuroimaging reports and neuroimages. Second, the performances of the ML models are limited by the number of annotated datasets. Additional training data are required to have a comprehensive comparison between the rule-based and ML systems. Third, the systems were only evaluated using datasets from 2 sites; the generalizability of the systems may be limited.

### Conclusions

We adopted a standardized data abstraction and modeling process to developed NLP techniques (rule-based and ML) to detect incidental SBIs and WMDs from annotated neuroimaging reports. Validation statistics suggested a high feasibility of detecting SBIs and WMDs from EHRs using NLP.

## Appendix

Multimedia Appendix 1 Gold standard development.

Multimedia Appendix 2 Supplementary result.

## Abbreviations

CNN: convolutional neural network
CT: computed tomography
EHR: electronic health record
IAA: interannotator agreement
IE: information extraction
MRI: magnetic resonance imaging
NLP: natural language processing
NPV: negative predictive value
PPV: positive predictive value
SBI: silent brain infarction
TMC: Tufts Medical Center
WMD: white matter disease
