# Supplementary material for: Natural Language Processing for the Identification of Silent Brain Infarcts From Neuroimaging Reports
Source: JMIR Med Inform. 2019 Apr 21;7(2):e12109. doi: 10.2196/12109 (PMC6524454; doi:10.2196/12109)
Supplement: Multimedia Appendix 1 [file medinform_v7i2e12109_app1.pdf]

Multimedia Appendix 1: Gold Standard Development

Gold Standard Development

The process of generating the gold standard reports followed a standardized data abstraction process with four major steps: data retrieval, cohort screening, guideline development, selection of reports and annotation (Figure 1).

Figure 1. Overview of gold standard creation process

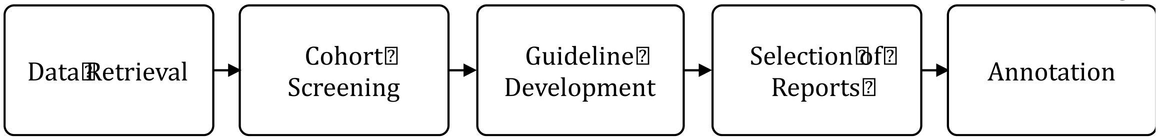

Data Retrieval

At TMC, the data comprised ICD-9 codes, CPT-4 codes, clinical notes and neuroimaging reports, and were obtained from three EHRs: General Electric Logician (outpatient general medicine), eClinicalWorks (outpatient specialties including neurology and neurosurgery), and Cerner Soarian (all inpatient encounters). At Mayo Clinic, the same types of data were retrieved from the Mayo Unified Data Platform (UDP).

Cohort Screening

A screening protocol, comprised of diagnosis codes and problem lists, was used to identify index neuroimaging reports (the patient's first MRI or CT in the EHR) for individual patients without clinically-evident stroke, transient ischemic attack (TIA), and dementia any time before or up to 60 days after the index imaging exam. The protocol was prototyped by a Vascular Neurologist (LYL) leveraging literature review(1, 2) and refined as a multi-disciplinary team effort including an internist (DMK), a neuroradiologist (PHL) and a radiologist (DFK). The final version of the protocol was vetted by both clinical and informatics teams from two sites. The protocol included both terminology codes and the corresponding text descriptions. For example, the text description of the code 434.10 is "cerebral embolism without cerebral infarction", which was used for text search. We excluded TIA because TIA is sometimes incorrectly assigned by clinicians as the diagnosis in the setting of transient neurologic symptoms and positive evidence of brain infarction on neuroimaging(3). Dementia was an exclusion criterion because of a projected future application of the NLP algorithm in identifying patients for comparative effectiveness studies or clinical trials for which both stroke and dementia could be outcomes of interest. To conduct cohort screening, an exclusion was applied when a patient has exclusionary ICD codes or his/her clinical note contained more than one type of keywords from the key terms list. An NLP system MedTagger(4) was utilized at Mayo Clinic to capture the mention from clinical notes. At TMC, this process was conducted through manual review. The screening result was validated manually by comparing the screened cohort with EMRs (clinicians: Mayo: PL, DI; TMC: LYL).

Guideline Development

A baseline annotation guideline and online educational videos were created to facilitate annotation guideline development. To develop the guideline, a preliminary annotation was performed individually by two senior radiologists, one senior neurologist and four medical residents. All annotators were asked to annotate the same 40 reports generated by both Mayo and Tufts. Feedback collected from the annotation was used to revise and update the guideline. The guideline includes task definition, annotation instruction, annotation concept and examples. The annotation task is to tag the findings of SBI and WMD lesion in both body (Findings) and summary (Impression and Assessment) sections of radiology reports. The definitions for SBI and WMD on both CT and MRI are mentioned in the guideline. For example, positive SBI findings on CT are defined as discrete, focal hypodense lesion greater than 3 mm in size conforming to a vascular distribution in the white matter, gray matter, or both(1, 2). For WMD, findings on MRI are defined as Confluent or poorly marginated T2 hyperintensities involving the white matter that do not meet criteria for brain infarction(1, 2). We defined likely but uncertain findings as SBI_Indeterminate. The annotation concept for SBI and WMD contains two categories: mentions of SBI and WMD related findings and attributes of the findings (e.g. Acuity for SBI and Grade for WMD).

Selection of Reports

At Mayo, 262,061 reports were obtained from Mayo EHR based on the CPT inclusion criteria. 4000 reports were randomly sampled for cohort screening. 910 were eligible for annotation after applying the ICD exclusion criteria (structured and unstructured). At TMC, 63,419 reports were obtained from TMC EHR based on CPT inclusion criteria. 12,092 reports remained after applying the ICD exclusion criteria (structured). 1000 reports were randomly selected for text screening. 773 reports were eligible for annotation.

Each site randomly selected 500 radiology reports (Total 1000) from the eligible samples (Mayo: 910, TMC: 773). 400 reports were randomly sample from the total 1000 reports (500 Mayo, 500 TMC) and duplicated for double reading in order to calculate inter-annotator agreement (IAA). Each resident was assigned 350 reports stratified by report type (CT, MR) and site (Mayo, TMC).

Annotation

Two third-year residents (KAK, MSC) from Mayo and two first-year residents (AOR, KN) from TMC performed the annotation. The annotation was organized into two phases. The first phase extended from the finalization of the preliminary guideline until the midpoint when one-half of the reports were annotated. The primary goal for the first phase was to identify new problems that were not captured in the sample data. This allowed the guideline to be more robust in accommodating new data. After the first phase, all problematic cases were reviewed by the two senior clinicians, and the guidelines were updated. The second phase annotation then commenced using the updated guidelines. A consensus meeting was organized to resolve all disagreements after the annotation. All conflicting cases were then adjudicated by the two senior clinicians. The annotation process was supervised by two senior clinicians (LKL, PL). All

the issues during the process were documented. The final gold standard corpus consisted of 1,000 annotated neuroimaging reports (500 TMC, 500 Mayo).

References

1. Fanning JP, Wesley AJ, Wong AA, Fraser JF. Emerging spectra of silent brain infarction. Stroke. 2014;45(11):3461-71.

2. Wardlaw JM, Smith EE, Biessels GJ, Cordonnier C, Fazekas F, Frayne R, et al. Neuroimaging standards for research into small vessel disease and its contribution to ageing and neurodegeneration. The Lancet Neurology. 2013;12(8):822-38.

3. Sorensen AG, Ay H. Transient ischemic attack: definition, diagnosis, and risk stratification. Neuroimaging Clinics. 2011;21(2):303-13.

4. Liu H, Bielinski SJ, Sohn S, Murphy S, Wagholikar KB, Jonnalagadda SR, et al. An information extraction framework for cohort identification using electronic health records. AMIA Summits on Translational Science Proceedings. 2013;2013:149.
