# Supplementary material for: Natural Language Processing for the Identification of Silent Brain Infarcts From Neuroimaging Reports
Source: JMIR Med Inform. 2019 Apr 21;7(2):e12109. doi: 10.2196/12109 (PMC6524454; doi:10.2196/12109)
Supplement: Multimedia Appendix 2 [file medinform_v7i2e12109_app2.pdf]

Multimedia Appendix 2: Supplementary Result

Effectiveness of Guideline Development

To assess the effectiveness of guideline development, the number of issues reported during the pre-guideline development and post-guideline development were documented and categorized into the following types of issues: modifier, medical concept, annotation rules, and linguistic. According to the Figure 1, there was a substantial decrease of the issues encountered during the annotation after the guideline was developed.

Figure 1. The Comparison of Annotation Issues in Two Phases

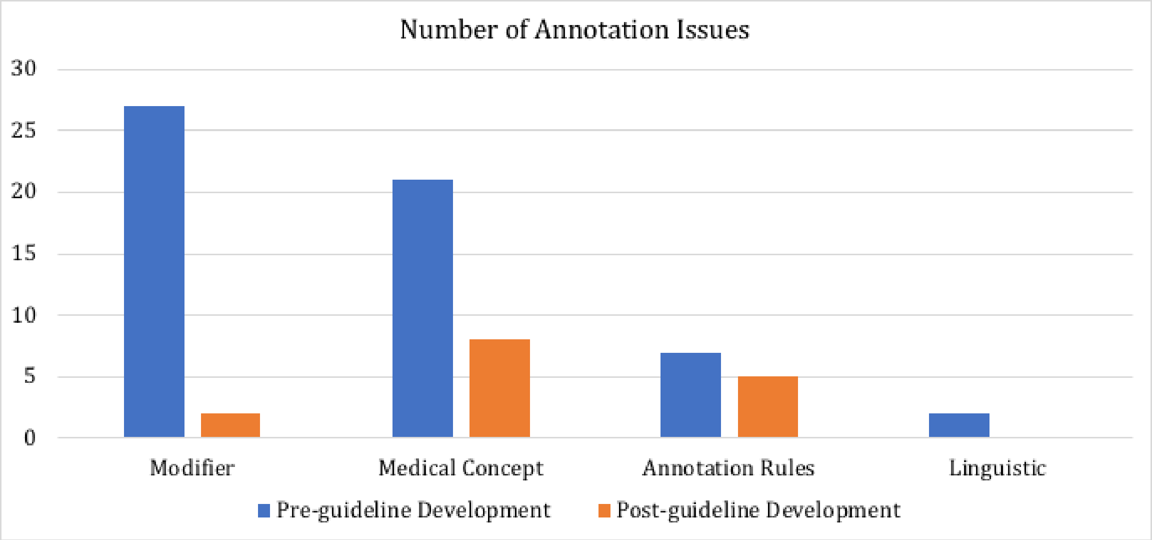

Age-specific Prevalence

Age-specific prevalence of SBI and of WMD was listed in Table 1. The overall rate of SBI was similar in our population compared to the literature (~15%)(1).

Table 1. Prevalence of SBI and of WMD in CT and in MRIs in 1000 Neuroimaging Reports Stratified by Age

	Mayo				Tufts			
	WMD	WMD%	SBI	%	WMD	WMD %	SBI	SBI%
**Age >= 50**								
	291	58.2	58	11.6	265	53.0	38	5.6
**Age >= 60**								
	210	63.8	48	14.6	209	62.6	32	7.5
**Age >= 70**								
	112	72.3	33	21.3	135	72.6	22	9.1
**Age >= 80**								
	39	73.6	14	26.4	52	83.9	12	16.1

Sublanguage Analysis

To prototype the rules and features for the IE system, a sublanguage analysis related to SBI and WMD in the 333 training reports was conducted to identify significant words and patterns associated with each condition. We leveraged Pointwise Mutual Information (PMI)[18] to measure the joint probability between the observed feature x and condition y with the probabilities of observing feature and condition independently. It is defined as

$$pmi(x,y) = log2\left(\frac{P(x,y)}{P(x)P(y)}\right) \quad (1)$$

The PMI was modified into the following equations to assess the inequality of likelihood of unigrams (one word) and bigrams (two adjacent words) in reports:

$$PMI(Gram, Class) = \log 2\,(N(Gram, Class) + \epsilon) * \left(log2\left(\frac{N(Gram,Class)+\epsilon}{N(Class)N}\right) - log2\left(\frac{N(Gram)}{N}\right)\right), where\ N(Gram, Class) \geq 1 \quad (2)$$

where N is the number of reports, N(*Gram*) is the number of reports having uni- or bigram *Gram*, N(*Gram, Cond*) is the number of reports with *Cond and* N *Gram*, and N(*Cond*) is the number of reports with condition *Cond*. Compared with Equation (1), Equation (2) was modified for penalizing low co-occurrence grams. This sublanguage analysis provided us with multiple textual biomarkers with strong associations with SBI and WMD. For example, the function classified “white matter” and “leukoaraiosis” as significant patterns associated with WMD. These textual markers were used for prototyping the initial keywords and rules.

The top ranked results from the PMI for rule prototyping is listed in Table 4.

Table 4. Top 15 Most Significant Unigram and Bigram Patterns Associated with SBI and WMD

	Unigram	Bigram
**SBI**		
	lacunar'; 'infarcts'; 'caudate'; 'thalamus'; 'corona'; 'radiata'; 'capsule'; 'old'; 'ganglia'; 'basal'; 'thalamic'; 'embolic'; 'lacune'; 'hemisphere'; 'subacute'	('lacunar', 'infarct'); ('lacunar', 'infarcts'); ('old', 'lacunar'); ('chronic', 'lacunar'); ('infarct', 'in'); ('infarcts', 'in'); ('small', 'chronic'); ('right', 'caudate'); ('an', 'old'); ('infarct', 'of'); ('small', 'old'); ('caudate', 'head'); ('occipital', 'lobes'); ('corona', 'radiata'); ('mild', 'diffuse')
**WMD**		
	periventricular'; 'subcortical'; 'ischemic'; 'scattered'; 'vessel'; 'chronic'; 'microangiopathy'; 'foci'; 'specific'; 'matter'; 'nonspecific'; 'leukoaraiosis'; 'white'; 'sequela'; 'finding'	('small', 'vessel'); ('vessel', 'ischemic'); ('white', 'matter'); ('subcortical', 'and'); ('periventricular', 'white'); ('foci', 'of'); ('scattered', 'foci'); ('and', 'periventricular'); ('non', 'specific'); ('chronic', 'microangiopathy'); ('of', 'chronic'); ('chronic', 'small'); ('a', 'non'); ('specific', 'finding'); ('but', 'likely')

Learning Rate

To ensure the convergence of the models, we plotted the learning curve for the CNN models.

Figure 1. Learning Curve on WMD data set for CNN model.

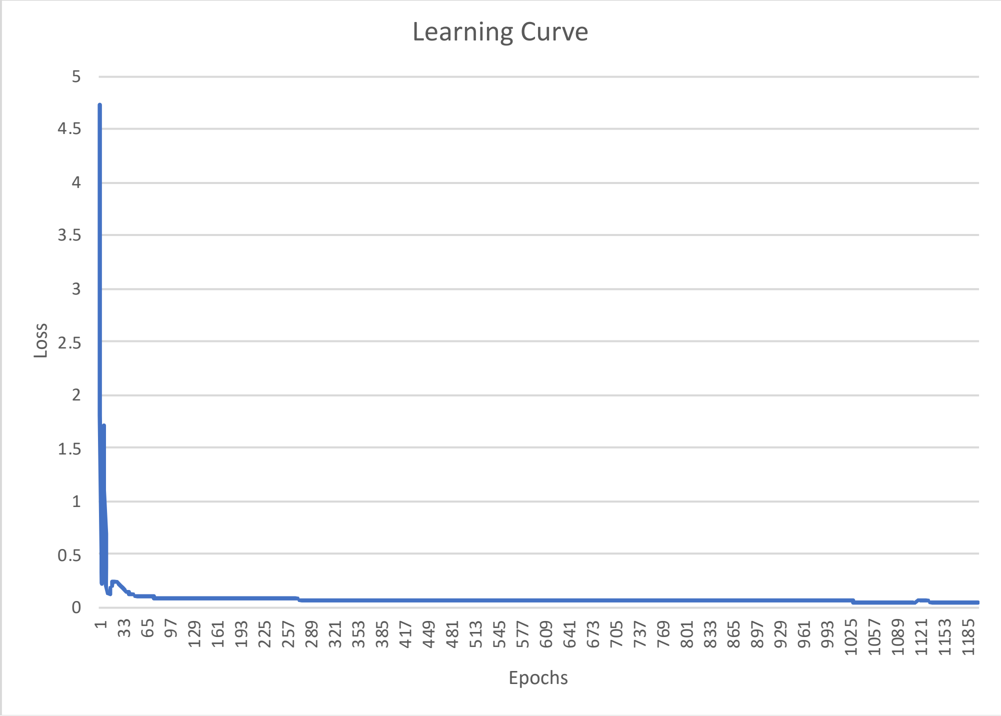

Figure 2. Learning Curve on SBI data set for CNN model.

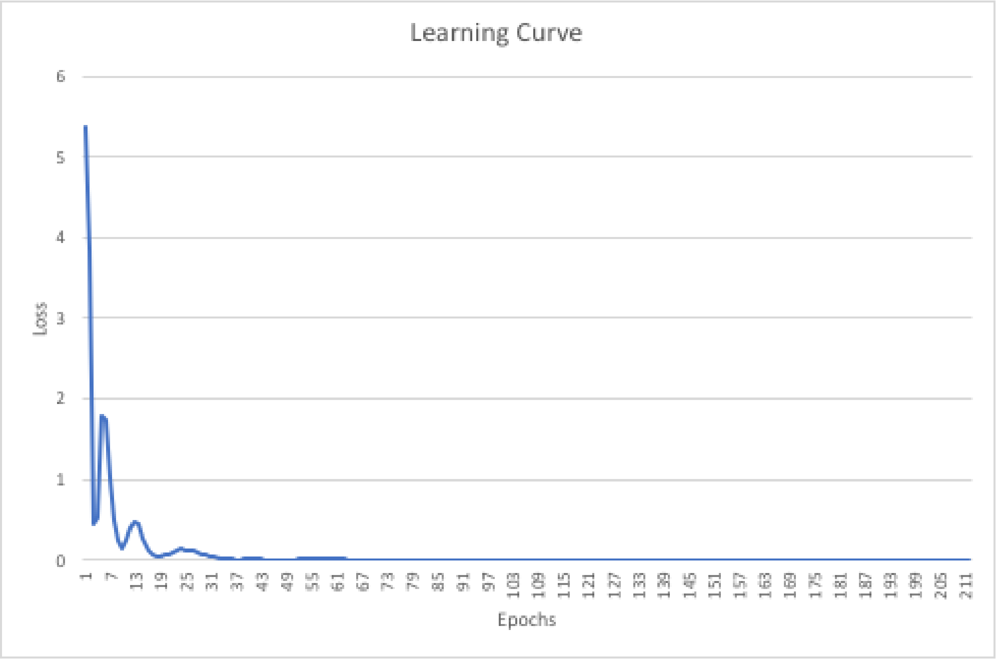

References

1. Fanning JP, Wong AA, Fraser JF. The epidemiology of silent brain infarction: a systematic review of population-based cohorts. BMC medicine. 2014;12(1):119.
